# Perinatal derivatives: How to best validate their immunomodulatory functions

**DOI:** 10.3389/fbioe.2022.981061

**Published:** 2022-09-14

**Authors:** Andrea Papait, Antonietta Rosa Silini, Maria Gazouli, Ricardo Malvicini, Maurizio Muraca, Lorraine O’Driscoll, Natalia Pacienza, Wei Seong Toh, Gustavo Yannarelli, Peter Ponsaerts, Ornella Parolini, Günther Eissner, Michela Pozzobon, Sai Kiang Lim, Bernd Giebel

**Affiliations:** ^1^ Department of Life Science and Public Health, Università Cattolica del Sacro Cuore, Rome, Italy; ^2^ Fondazione Policlinico Universitario “Agostino Gemelli” IRCCS, Rome, Italy; ^3^ Centro di Ricerca E. Menni, Fondazione Poliambulanza Istituto Ospedaliero, Brescia, Italy; ^4^ Department of Basic Medical Sciences, Laboratory of Biology, Medical School, National and Kapodistrian University of Athens, Athens, Greece; ^5^ Department of Women and Children Health, University of Padova, Padova, Italy; ^6^ Laboratorio de Regulación Génica y Células Madre, Instituto de Medicina Traslacional, Trasplante y Bioingeniería (IMeTTyB), Universidad Favaloro-CONICET, Buenos Aires, Argentina; ^7^ School of Pharmacy and Pharmaceutical Sciences, Trinity College Dublin, Dublin, Ireland; ^8^ Trinity Biomedical Sciences Institute, Trinity College Dublin, Dublin, Ireland; ^9^ Trinity St. James’s Cancer Institute, Trinity College Dublin, Dublin, Ireland; ^10^ Department of Orthopaedic Surgery, Yong Loo Lin School of Medicine, National University of Singapore, Singapore, Singapore; ^11^ Laboratory of Experimental Hematology, Vaccine and Infectious Disease Institute (Vaxinfectio), University of Antwerp, Antwerp, Belgium; ^12^ Systems Biology Ireland, School of Medicine, Conway Institute, University College Dublin, Dublin, Ireland; ^13^ Institute of Medical Biology and Institute of Molecular and Cell Biology, Agency for Science, Technology and Research, Singapore, Singapore; ^14^ Institute for Transfusion Medicine, University Hospital Essen, University of Duisburg-Essen, Essen, Germany

**Keywords:** perinatal derivatives, mesenchymal stromal cells, functional assays, mechanisms of action, extracellular vesicles, exosomes, microvesicles, immunomodulation

## Abstract

Perinatal tissues, mainly the placenta and umbilical cord, contain a variety of different somatic stem and progenitor cell types, including those of the hematopoietic system, multipotent mesenchymal stromal cells (MSCs), epithelial cells and amnion epithelial cells. Several of these perinatal derivatives (PnDs), as well as their secreted products, have been reported to exert immunomodulatory therapeutic and regenerative functions in a variety of pre-clinical disease models. Following experience with MSCs and their extracellular vesicle (EV) products, successful clinical translation of PnDs will require robust functional assays that are predictive for the relevant therapeutic potency. Using the examples of T cell and monocyte/macrophage assays, we here discuss several assay relevant parameters for assessing the immunomodulatory activities of PnDs. Furthermore, we highlight the need to correlate the *in vitro* assay results with preclinical or clinical outcomes in order to ensure valid predictions about the *in vivo* potency of therapeutic PnD cells/products in individual disease settings.

## Introduction

The first report that cells from perinatal tissues may provide a promising source for novel cellular therapies was published in 2004 (Bailo et al., 2004). In this pioneering study it was shown that mesenchymal stromal cell (MSC)-like human amnion and chorion cells obtained from human term placenta had the capability to suppress lymphocyte responsiveness *in vitro*. Furthermore, these cells could engraft into neonatal pigs and rats without being rejected (Bailo et al., 2004). The broad availability of placental tissues as biological waste products; the ease in isolating and expanding perinatal cells from various regions of the placenta; and the cells’ potent immunomodulatory properties, have attracted increasing research interest developing the potential of placental cells and their secretome for treating a variety of diseases, especially those characterized by an inflammatory disbalance (Silini et al., 2020). To harmonize research in the field of perinatal derivatives (PnDs), the First International Workshop on Placenta-derived Stem Cells was held in Brescia, Italy in 2007. In this workshop four major regions of the fetal placenta were discussed as possible sources for therapeutically relevant stem and progenitor cells, namely the amniotic epithelial, the amniotic mesenchymal, the chorionic mesenchymal, and the chorionic trophoblastic tissues (Parolini et al., 2008). Since then, a variety of additional cell types, including endothelial cells, have been also isolated from other perinatal tissues and studied for their therapeutic functions. To harmonize the nomenclature and the criteria for the definition of cells isolated from different perinatal tissues and as an output of the European Union Cooperation in Science and Technology (COST) Action International Network for Translating Research on Perinatal Derivatives into Therapeutic Approaches (SPRINT), we recently published an update on the nomenclature and criteria (Silini et al., 2020). Despite most PnDs, including perinatal cells themselves as well as products obtained from their secretome, have already demonstrated huge therapeutic potential in various preclinical studies, no uniformity and/or standardization in potency testing exists so far. In this manuscript we will highlight the importance of testing the therapeutic potency of PnDs using appropriate functional assays and introduce several of the challenges that need to be considered for translating PnDs into the clinics.

As there is extensive experience with MSC products most commonly obtained from adult material, mainly adult bone marrow and fat tissue (Moll and James. 2019; Ringdén et al., 2022), we first summarize key aspects and challenges of the therapeutic MSC field, and highlight the need for robust assays to test the therapeutic potency of adult MSC and PnD products. In addition to pro-regenerative functions, PnDs like adult MSCs are known for their ability to modulate immune responses. Accordingly, in the second part of this manuscript, using the example of functional assays exploring T cell and monocyte/macrophage activities, we provide various considerations for setting up appropriate functional assays for evaluating the therapeutic potency of MSC and PnD products.

### Lessons from the adult MSC field

Although our main focus is PnDs, the challenges identified in clinical translation of MSCs has provided many learning points that are of substantial relevance to the development of envisioned PnD therapies. Thus, for clarity it is appropriate to briefly introduce the MSC field and some of its current limitations.

Historically, non-hematopoietic therapeutic stem cell research began with the discovery of MSCs raised from adult bone marrow cells in the 1960s (Friedenstein et al., 1968), and the demonstration of their multi-lineage potential at the turn of the millennium (Pittenger et al., 1999). Considering their differentiation potential far beyond the mesenchymal lineage (Munoz-Elias et al., 2003; Pittenger and Martin 2004), MSCs quickly emerged as a promising stem cell entity for regenerative approaches, either in the autologous or allogeneic setting. Connected to proposed allogeneic MSCs applications, their interaction with different allogenic immune cells has been studied in detail.

Contrary to the initial expectations, upon administration *in vivo*, allogenic MSCs are not acutely destroyed by the immune system, but display a strong modulating function on various immune cells by suppressing their pro-inflammatory activities and inducing their regulatory, i.e., their tolerogenic, functions (Bartholomew et al., 2002; Di Nicola et al., 2002; Meisel et al., 2004). Consequently, MSCs have been increasingly tested for their immunomodulatory capability in preclinical models, as well as in a number of different clinical studies (Moll and James. 2019; Ringdén et al., 2022). One of the first administrations of MSCs in a patient with inflammatory disbalance was performed by Le Blanc and others when they succesfully treated a steroid refractory Graft-versus-Host Disease (GvHD) patient with adult bone marrow derived MSCs (Le Blanc et al., 2004).

In addition to bone marrow, MSCs have been isolated from various adult tissues, including fat (Moll and James. 2019). They have also been isolated from perinatal tissues, namely placenta, umbilical cord tissue and umbilical cord blood (Silini et al., 2020) and from the human second trimester amniotic fluid (AF-MSCs) (Roubelakis et al., 2007; Roubelakis et al., 2011; Legaki et al., 2016). Irrespective of their origin, MSCs obtained from all tissues share some common features that have been defined as *bona fide* criteria for MSCs by the International Society of Cell and Gene Therapy in 2006 (Dominici et al., 2006). Specifically, they grow as plastic-adherent cells and possess the ability to differentiate to various lineages including osteogenic, adipogenic and chondrogenic lineages. Furthermore, MSCs express characteristic cell surface antigens including CD73, CD90 and CD105 and they lack expression of hematopoietic and endothelial cell specific antigens including CD14, CD45 and CD34, CD11b and CD79a, or CD19 and HLA-DR (Dominici et al., 2006). Due to their multipotency they were initially defined as mesenchymal stem cells, however, over the years it became clear that despite their multipotency they lack stem cell features and thus, have been renamed in multipotent stromal cells (Caplan 1991; Viswanathan et al., 2019).

To date, MSCs, mainly raised from adult tissues, have been registered in more than 1,400 clinical trials, either in regenerative settings or as immunomodulatory agents (clinicaltrials.gov). As exemplified by the MSC treatment of GvHD patients, many studies including a phase III clinical trial reported therapeutic efficacy of MSC therapies in GvHD patients, while others also including a phase III clinical trial failed to show efficacy (Baron and Storb 2012; Galipeau 2013; Kurtzberg et al., 2020a; Kurtzberg et al., 2020b; Kebriaei et al., 2020). Explaining the current controversy, over the years it has become clear that, despite some common features, MSCs represent a heterogenous cell entity with tissue-specific and intra-individual differences (Phinney et al., 1999; Vogel et al., 2003; Phinney 2012; Radtke et al., 2016).

Although known for several years now, discussions about the impact of this heterogeneity on the clinical outcome of MSC therapies have just begun (Dunn et al., 2021; Galipeau et al., 2021; Krampera and Le Blanc 2021). Among others, the relevance of differences in the expression level of the clotting cascade inducing tissue factor (TF) among MSCs of different origins emerged as a critical discussion point. To this end, placental and fat MSCs express higher levels of TF than BM-MSCs and thus may provide higher thromboembolic complications risks following MSC administration than that of BM-MSCs (Moll et al., 2022). As a consequence of such potential functional differences, regulatory authorities are increasingly requesting clarity on the role of cell heterogeneity and functionality. It is expected that, in the future, such clarity will be sought for other non-hematopoietic stem/progenitor cell products. In this context it is worth highlighting that this issue was critical in the US Food and Drug Administration (FDA) evaluation of the commercial MSC product remestemcel-L, which had shown efficacy in suppressing paediatric acute GvHD when evaluated in a single armed phase III clinical trial (Kurtzberg et al., 2020a; Kurtzberg et al., 2020b). The US FDA opined that as the critical quality attributes (CQAs) did not correlate with clinical effectiveness and/or *in vivo* potency/activity, the clinical effectiveness of individual lots of remestemcel-L being produced from graft material of varying donors was not adequately controlled. Without a proper CQA strategy, substantial functional heterogeneity that often is observed between independent MSC batches, especially when derived from different donors, may not be detectable. Consequently, in October 2020 the US FDA declined the approval of remestemcel-L for the treatment of paediatric acute GvHD in the United States (https://www.fda.gov/media/140988/download). Thus, it is evident that potency assays based on CQAs that are linked to a clearly defined mechanism of action (MoA), and/or CQAs that have a demonstrated relationship with clinical efficacy are critical to the successful translation of cellular products into the clinics.

Similar to MSCs, perinatal MSC products and other PnDs are complex biological products with multimodal *in vivo* activity that are likely to vary in donor dependent manners. Furthermore, such activities are highly affected by cell seeding and expansion conditions, as well as the duration of culture, both in number of passages or the duration between passaging. Thus, it is timely to consider and re-evaluate the reliability of existing functional assays that are frequently used for the characterization of PnDs, especially in view of their ability to link *in vitro* regenerative/immunomodulatory properties with clinical potency.

As such, for cell-based assays it will be important to determine specific PnD attributes that are being measured. If these attributes are involved in a clearly defined therapeutic MoA by the PnD against a specific disease, these attributes could be qualified as CQAs and the assays for these CQAs could be eventually used as potency assays to predict and ensure potency of individual lots of given PnD preparations. Before discussing functional assays for predicting potency in greater detail, basic concepts of the MoA of MSCs and PnDs will be discussed next.

### MSCs mediate many therapeutic effects *via* their secretome

Administered MSCs were initially considered to home into affected tissues and to replace lost cell types in regenerative approaches or to modulate immune responses by direct cell-to-cell contacts in inflamed tissues. Upon studying their biodistribution, however, it was recognized that most of systemically administered MSCs embolise the lungs and are rarely recovered in affected tissues (Gao et al., 2001; Schrepfer et al., 2007; Lee et al., 2009). Therefore, it was postulated that MSCs may act in a paracrine, rather than in a cellular manner (Caplan 2017). Indeed, in myocardial infarction models, administration of their conditioned media or encapsulated MSCs induced comparable therapeutic effects to those achieved with systemically applied MSCs (Gnecchi et al., 2005; Gnecchi et al., 2006; Timmers et al., 2007). Thus, it became clear that MSCs mediate tissue repair in many applications through their secretome, particularly EVs (Bruno et al., 2009; Lai et al., 2010). Indeed, EVs have already shown clinical improvement in an acute GvHD patient, in chronic kidney disease patients, in a cochlear implant patient and in many animal models (Kordelas et al., 2014; Nassar et al., 2016; Warnecke et al., 2021). Despite these applications revealed positive effects of the applied MSC-EV products, variable activities and inter-donor heterogeneity among independent MSC-EV products should still be expected. Indeed, as shown at the example of murine models for GvHD, ischemic stroke and Niemann’s Pick Type C, independent MSC-EV preparations can differ in their ability in suppressing respective disease symptoms (Madel et al., 2020; Wang et al., 2020; Van Hoecke et al., 2021). Similarly, with the aim to translate PnDs, including EVs derived thereof, into a clinical setting, it is strongly recommended to establish a suitable potency testing and reliable functional analysis toolbox for PnDs from the outset.

## Assays for the assessment of immunomodulatory PnD activities

It is generally assumed that, as for adult MSCs, one of the major PnD associated activities is their capability to modulate immune responses (Börger et al., 2017; Silini et al., 2020). Consequently, their immunomodulatory activities are frequently investigated in various *in vitro* assays, primarily on T cells or on macrophages. To this end, a huge variety of protocols for simple *in vitro* assays and subsequent read-out strategies have been developed. However, not all of these assays monitor the same immunomodulatory activity. Furthermore, not all monitored activities are involved in the mechanism by which cells exert their therapeutic effects for an indicated disease. In fact, in the past, most *in vitro* potency assays have failed to reliably and reproducibly predict the clinical effectiveness of administered MSCs (Galipeau et al., 2016). Thus, it is important that potency assays intended to predict the therapeutic function of a PnD measure the activities that are of direct relevance to the PnD’s MoA for a specific disease. Without favoring any specific procedure, we feel it is important to discuss assay relevant parameters and potential caveats of respective assays and potential read-out strategies. Of note, this review is part of a quadrinomial series on functional assays for validation of PnDs, spanning biological functions, such as immunomodulation, anti-inflammation, anti-microbial/anti-cancer, wound healing, angiogenesis and regeneration.

### T cell assays

T lymphocytes are the main component of the adaptive immune response and have an extremely high capacity to discriminate between self and non-self. In fact, they express a series of highly polymorphic receptors that allow them to actively respond to antigens presented either by antigen presenting cells or by infected cells. This type of response triggers both CD4 T helper and cytotoxic CD8 T lymphocytes (Mueller et al., 2013; Kumar et al., 2018). Furthermore, both CD4 and CD8 T lymphocytes are capable of developing immunological memory, a feature typical of adaptive immunity (Mueller et al., 2013; Kumar et al., 2018). Coupled to their ability to discriminate between self and non-self, T lymphocytes are the main actors in allograft rejection; they also play a relevant role in autoimmunity (Marino, Paster et al., 2016; Khan and Ghazanfar 2018). However, not all T lymphocytes mediate defense functions, a proportion of them, especially the regulatory T cells mediate tolerogenic functions that are required during the whole course of pregnancy and other developmental and regenerative processes (Weirather et al., 2014; Boothby et al., 2020; Fung et al., 2020; Green et al., 2021). As important as these regulatory T cell functions are, regulatory T cells also promote tumor growth and have been identified as promising targets in anti-tumor therapies (Paluskievicz et al., 2019; Bai et al., 2020; Seed et al., 2021).

Many degenerative and acute diseases including GvHD, ischemic stroke, sepsis and COVID-19 are accompanied by uncontrolled pro-inflammatory reactions, regularly also involving T cell effector responses (Hill 2009; Nakamura et al., 2020). Upon administration of potent PnD or adult MSC products that promote regeneration or improvement of acute disease symptoms, respectively, pathology associated T cell effector responses get suppressed *in vivo* and frequently are converted into regulatory T cell responses (Balza et al., 2016; De Biasi et al., 2021; Strobl et al., 2021). Furthermore, perinatal and adult MSC products can convey immunomodulatory activities in different autoimmune disease models, highlight their ability to reduce Th1/Th17 imbalances and to trigger T cell polarization towards regulatory T cell functions (Sun et al., 2009; Obermajer et al., 2014; Parolini et al., 2014; Tsai et al., 2014; Wang D et al., 2014; Wang H et al., 2014; Wang et al., 2017; Ma et al., 2019).

Coupled to such observations, it is broadly assumed that one of the central MoA attributes of PnD and adult MSC products is their ability to suppress T effector and to induce regulatory T cell functions. Consequently, many groups have started to study impacts of PnD and adult MSC products on T cells in a variety of different T cell assays.

Fundamentally, T cell assays can be categorized by whether they use primary or cell line derived T cells, such as Jurkat cells. Even though the usage of cell lines allows a higher degree of standardization, they regularly contain an array of different genetic mutations some of which can affect the reactivity of respective cells on environmental factors and thus change some of their key functions (Khan and Ghazanfar, 2018). For example, although Jurkat cells are widely used to study T cell receptor (TCR) signaling (Abraham and Weiss, 2004), they in contrast to primary T cells poorly respond to immunomodulatory signals including those of MSC products (Zhang et al., 2017).

**FIGURE 1:**
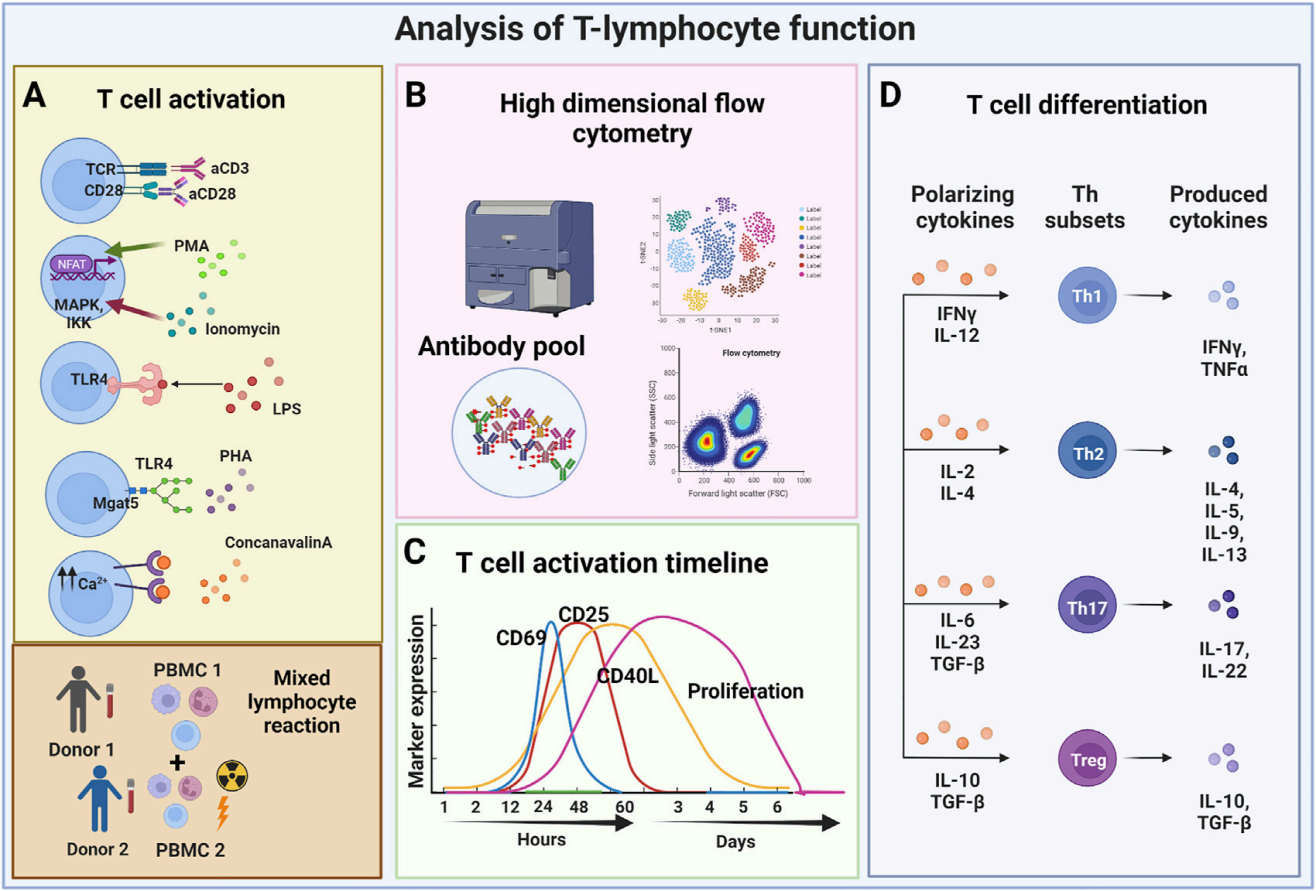
Analysis of T-lymphocyte function. **(A)** T lymphocytes can be activated using various stimuli including monoclonal antibodies (stimulation with anti-CD3 anti-CD28), and mitogens, such as PMA frequently used in combination with ionomycin. Other modes of stimulation include the use of lipopolysaccharides that triggers TLR4 activation (thus mimicking the bacterial stimulus), and causing the release of PHA that induces the recruitment of TCRs consequently activating T lymphocytes. Finally, T lymphocytes can also be stimulated by ConA, which causes the release of intracellular Ca^2+^ that triggers the calcium cascade, and by mixed lymphocyte reactions which are based on the allogeneic response determined by HLA mismatching between two different donors. **(B)** Depending on the considered mechanism of action, different readout methods are used. Flow cytometry can perform in-depth immune-phenotype analyses. **(C)** Various markers can be used to analyze T cell activity in given assays, some being selectively expressed at specific timepoints following T cell activation. **(D)** The functional polarization of T lymphocytes can be triggered with different combinations of cytokines towards different CD4 Th subsets or towards different CD8 memory T cell subsets. Cytokine analyses provide important information about resulting T cell functions. (Created with BioRender.com).

Assays using primary T cells can be subdivided in two additional main categories, those which are based on purified T cells (either CD3, CD4, or CD8 T cells) or those which use mixtures of cells, typically peripheral blood mononuclear cells (PBMCs). In virtually all T cell assay variants, T cells are experimentally activated. As a range of different T cell activating stimuli are used, the variety of available T cell assays is further multiplied. Consequently, comparison of the effects of PnD and adult MSC products on T cell proliferation becomes difficult when different stimuli are used. Frequently T cells are activated by the addition of mitogens, such as the lectin phytohemagglutinin (PHA) (Ceuppens et al., 1988), Ionomycin (Chatila et al., 1989; Hou et al., 2018) usually in combination with tumor promoting agents, such as phorbol 12-myristate 13-acetate (PMA) (Lehnert et al., 2014; Hou et al., 2018) or concanavalin A (ConA) (Palacios, 1982; Arneth, 2010); or by pro-inflammatory bacterial products such as lipopolysaccharides (LPS) (Zanin-Zhorov et al., 2007). Another strategy is based on T cell activating antibodies that are typically directed against the T cell surface molecules CD3 and/or CD28 (Trickett and Kwan, 2003; Jiao et al., 2019) (Figure 1A). Furthermore, T cells can be activated by allogenic cells, typically in mixed lymphocyte reaction (MLR) assays (Tomonari, 1980; Yang et al., 2009). Here, T cells of at least one given donor are co-cultured with immune cells of other allogenic donors, or with specific allogenic T cell response-inducing cell line cells. In classical MLR assays, T cell stimulating cells are regularly irradiated to inhibit their own proliferation (Sasazuki et al., 1976) (Figure 1A). However, assays have also been developed in which mononuclear cells, including T cells of multiple donors, have been combined for effective allogenic T cell activation (Pachler et al., 2017; Madel et al., 2020) (Table 1). Additional critical parameters in such assays are the numbers of seeded cells, the cells’ seeding densities, the choice of the cell culture containers, the assay duration, and the cell culture media including their supplements, e.g., serum and/or recombinant cytokines.

**TABLE 1:** Type of stimuli used in T-cell assays.

Population	Stimulation	Observed effect	Reference
human T lymphocytes	phytohemagglutinin (PHA)	PHA as a mitogen induces T lymphocyte proliferation	Ceuppens et al. (1988)
purified resting human T lymphocytes	ionomycin	ionomycin induces the proliferation of T lymphocytes	Chatila et al. (1989)
peripheral blood mononuclear cells (PBMC)	ionomycin + phorbol 12-myristate 13-acetate (PMA)	stimulation of T lymphocyte proliferation	Hou et al., (2018), Lehnert et al. (2014)
Total PBMC/purified T lymphocytes	concanavalin A (ConA)	stimulation of T lymphocyte proliferation	Arneth, (2010), Palacios (1982)
purified T lymphocytes (human and mouse)	lipopolysaccharides (LPS)	stimulation of T lymphocyte proliferation by triggering Toll-like receptor 4 (TLR4)	Zanin-Zhorov et al. (2007)
PBMC/purified T lymphocytes	anti-CD3/anti-CD28 antibodies	T lymphocyte proliferation due to activating antibodies that are typically directed against the T cell surface molecules CD3 and/or CD28	Jiao et al. (2019), Trickett and Kwan (2003)
PBMC (responder) vs. g-irradiated PBMC (stimulator)	mixed lymphocyte reaction (MLR) assay	proliferation of T lymphocytes due to HLA mismatching that triggers the activation of the responder PBMC, while the stimulator gamma-irradiated PBMC do not proliferate	Tomonari (1980), Yang et al. (2009)
PBMC	multidonor mixed lymphocyte reaction (mdMLR) assay	proliferation and activation of T lymphocytes due to HLA mismatching that triggers mutual activation of PBMC of the different donors	Madel et al. (2020), Pachler et al. (2017)

For the selection of an appropriate T cell assay, it must be considered whether PnD or adult MSC products may act directly or indirectly on T cells. For example, it has been reported that MSC-EVs do not directly act on T cells; rather, they modulate the biology of co-cultured monocytes/macrophages by altering their secretome (Zhang et al., 2014; Zhang B. et al., 2018). Furthermore, dosing should be carefully considered. Ideally physiologically relevant concentrations of PND and adult MSC products should be applied in the functional assays. However, because MSC products may act in a complex cascade with different cellular targets (Gimona et al., 2021), it might be that *in vivo* their therapeutic activities are much more exponentiated than in given *in vitro* assays. Thus, *in vitro* experiments may require higher product doses than related *in vivo* applications.

The mode of activation of T cells is critical for their response to PnD or adult MSC product modulation (Kronsteiner et al., 2011). Mitogens act on several signaling pathways, some of which bypass the direct triggering of TCR and co-stimulatory molecules. This is the case, for example, with ionomycin, which induces intracellular calcium release and subsequent phospholipase C activation, hydrolysis of phosphoinositides and activation of Protein Kinase C (PKC) (Hossain et al., 2007). As mentioned before, ionomycin is usually used in combination with PMA, which is a specific activator of PKC, thus exerting a synergistic action (Lehnert et al., 2014; Hou et al., 2018). In contrast, ConA is an activator of the transcription factors Nuclear Factor of Activated T cells (NFAT), a family of transcription factors that are important in the development and function of the immune system, including TCR engagement (Bemer and Truffa-Bachi, 1996). PHA, can lead to rapid T cell activation by specifically binding to the Alpha-1,6-Mannosylglycoprotein 6-Beta-N-Acetylglucosaminyltransferase (Mgat5) receptor expressed on the surface of T lymphocytes, thus triggering different signalling pathways that, in turn, induce the recruitment of TCRs and the activation of T lymphocytes (Demetriou et al., 2001) (Table 2 and Figure 1A). It has been estimated that the recruitment and clustering of approximately 8,000 TCRs is required to lead to the activation of T lymphocytes. However, it should also be emphasized that this stimulation mode is very different from the physiological activation obtained following stimulation with anti-CD3 and anti-CD28 antibodies or by allogenic stimulation. This number is significantly reduced when stimulation is performed with antibodies against CD3 and CD28 (Viola and Lanzavecchia, 1996). Thus, mitogen activation does not reflect the physiological situation and alters normal T cell functions including their differentiation and maturation capabilities from naïve to effector cells (Duarte et al., 2002; Maus et al., 2002). Consequently, activities recorded by such assays may not reflect the *in vivo* potency of PND and adult MSC products.

**TABLE 2:** Mechanism of action of the different stimuli and possible readouts.

Population	Experimental procedure	Observed effect	Reference
naive Murine T-Cells	stimulation with ionomycin	Ionomycin induces intracellular calcium release and subsequent phospholipase C activation, hydrolysis of phosphoinositides and activation of Protein Kinase C (PKC)	Hossain et al. (2007)
PBMC	stimulation with PMA	PMA is a specific activator of PKC thus exerting a synergistic action	Hou et al. (2018), Lehnert et al. (2014)
purified mouse T lymphocytes	stimulation with ConA	ConA is an activator of Nuclear Factor of Activated T cells (NFAT), a family of transcription factors that are important in the development and function of the immune system, including TCR engagement	Bemer and Truffa-Bachi (1996)
mouse naive purified T lymphocytes	stimulation with PHA	PHA can lead to rapid T lymphocyte activation by specifically binding to the alpha-1,6-mannosylglycoprotein 6-beta-N-acetylglucosaminyltransferase (Mgat5) receptor expressed on the surface of T lymphocytes, thus triggering different signalling pathways that, in turn, induce the recruitment of TCRs and the activation of T lymphocytes	Demetriou et al. (2001)
purified T lymphocytes	staining with carboxyfluorescein diacetate succinimidyl ester (CFSE) or PKH	the proliferation rate of activated T lymphocytes is typically analysed after staining with fluorescent dyes, e.g. CFSE or PKH dyes, whose intensities decrease after cell division	Tario et al. (2011)
total PBMC/ purified T lymphocytes	evaluation of different activation markers by flow cytometry	cell surface molecules are established as being upregulated on activated T lymphocytes: these include the early activation marker CD69 and late activation markers, such as the IL-2 receptor (CD25) and the intercellular adhesion molecule 1 (ICAM-1; CD54)	Lindsey et al. (2007), Sancho et al. (1999)
PBMC	cytokine evaluation	production and release of cytokines as well as polarisation towards specific subsets indicate a functional change in T lymphocytes as a result of the stimulation received. By using bivalent antibodies, such changes can be monitored using flow cytometry. More frequently, however, the cytokine content in conditioned media is analysed by a conventional cytokine analysis method. Elispot assays, where cells are cultured on a membrane, allow quantification of cells secreting specific cytokines	Bueno et al. (2001)
PBMC/ purified T lymphocytes	analysis of Th subset polarization	PnDs are able to influence the differentiation of purified naïve T lymphocytes stimulated with monoclonal anti-CD3 and/or anti-CD28 antibodies, converting T lymphocyte development under Th1 or Th17 differentiation conditions towards development of CD4+ Th2 T lymphocytes	Liu et al. (2014), Pianta et al. (2015), Krampera et al. (2003)

A common hallmark of the various type of T cell assays is that the T cells become activated and proliferate within these assays (Iritani et al., 2002; Obst, 2015). Coupled to the activation, T cell gene expression profile and cytokine secretion changes. Activation can also trigger the differentiation of naïve T lymphocytes as well as the polarization of naïve T cells towards different T effector cell subsets (Luckheeram et al., 2012). Different read outs are used to analyse T cell activation and proliferation. The proliferation rate of activated T cells is typically analysed after staining with fluorescent dyes, e.g., carboxyfluorescein diacetate succinimidyl ester (CFSE) or PKH dyes, whose intensities following labelling get mainly reduced by cell divisions (Tario et al., 2011). The fluorescence intensity of labelled cells is regularly monitored by flow cytometry (Figure 1B). Depending on the purpose of the experiment, more complex cell surface analyses can be performed. For example, by using a selection of different antibodies, the resolution of such assays can be increased in order to study cell proliferation of specific T cell subsets. Flow cytometry can be also informative without exploring the proliferation history of respective T cells. For example, activation of T cells results in their cell growth being accompanied by changes in their light scattering features. Furthermore, a couple of different cell surface molecules are established as being upregulated on activated T cells. These include the early activation marker CD69 (Lindsey et al., 2007) and later activation markers, such as the IL-2 receptor (CD25) and the intercellular adhesion molecule 1 (ICAM-1; CD54) (Sancho et al., 1999) (Table 2 and Figure 1C).

Although T cell proliferation assays or analyses of the T cell activation status are frequently used for studying the impacts of PnD and adult MSC products, the expectation that all therapeutically relevant products result in a suppression of T cell proliferation or in efficient inactivation of T cells might not necessarily be true and, indeed, has been challenged by several groups. For example, it has been shown that following primary activation of CD69 via the canonical NFκB signalling pathway, MSCs can promote its expression in a non-canonical manner. In the absence of MSCs, at a later stage, canonical NFκB signalling apparently contributes to the reduction of CD69 expression. Thus, canonical NFκB signalling plays a dual role, i.e. at the early stage it activates and at a later stage it terminates the expression of CD69. Apparently, the later function can be suppressed by MSCs (Saldanha-Araujo et al., 2012) (Table 2).

Following activation, T cells also change their cytokine secretion. Indeed, the production and release of cytokines as well as polarisation towards specific subsets are indicative for a functional change in T lymphocytes as a result of the stimulation received. By using bivalent antibodies, such changes can be monitored flow cytometrically (Bueno et al., 2001). More frequently, however, the cytokine content in conditioned media is analyzed by a conventional cytokine analysis method. Elispot assays, in which cells are cultured on a membrane, allow quantification of cells secreting specific cytokines. Notably, if T cells are cultured in the presence of other immune cells, changes in the concentration of the cytokines in cell supernatants may also be caused by non-T cells (Table 2).

The variability in performing T cell activation assays is amplified by the PnD and adult MSC products to be tested. If the function of cellular products should be evaluated, cell culture conditions need to be used which are permissive for the cells to be explored and the T cell containing cell fraction. In contrast, EV and other secretome products can be added to T cells cultured under optimal growth conditions.

The activation of T lymphocytes following the use of different stimuli is a fundamental prerequisite to not only trigger proliferation, but also for the differentiation of naïve T lymphocytes and their polarization towards effector subsets. In fact, PnD and adult MSC products have been reported to affect the differentiation of naïve T lymphocytes towards effector and memory subsets (Liu et al., 2014; Pianta et al., 2015). PnDs were also shown to influence the differentiation of purified naïve T lymphocytes, especially, they could convert T cell development under Th1 or Th17 differentiation conditions towards development of CD4^+^ Th2 T lymphocytes (Krampera et al., 2003; Liu et al., 2014) (Figure 1D). Such impacts on T cell polarization and differentiation can be stimulus dependent, for example MSC secretome products could polarize T cells towards Tregs when stimulated by allogenic antigen presenting cells (APCs), but not if T cells were activated anti-CD3 and anti-CD28 antibodies (Zhang S. et al., 2018) (Table 2 and Figure 1D).

We are aware there are many other parameters that essentially influence the outcome in T cell assays and that would be worthy of discussion, e.g., the co-incubation time of PnD and adult MSC products and T cells. While further refining such assays, there might even be other parameters we are not yet aware of that could essentially affect given readouts.

### Monocyte and macrophage assays

**FIGURE 2:**
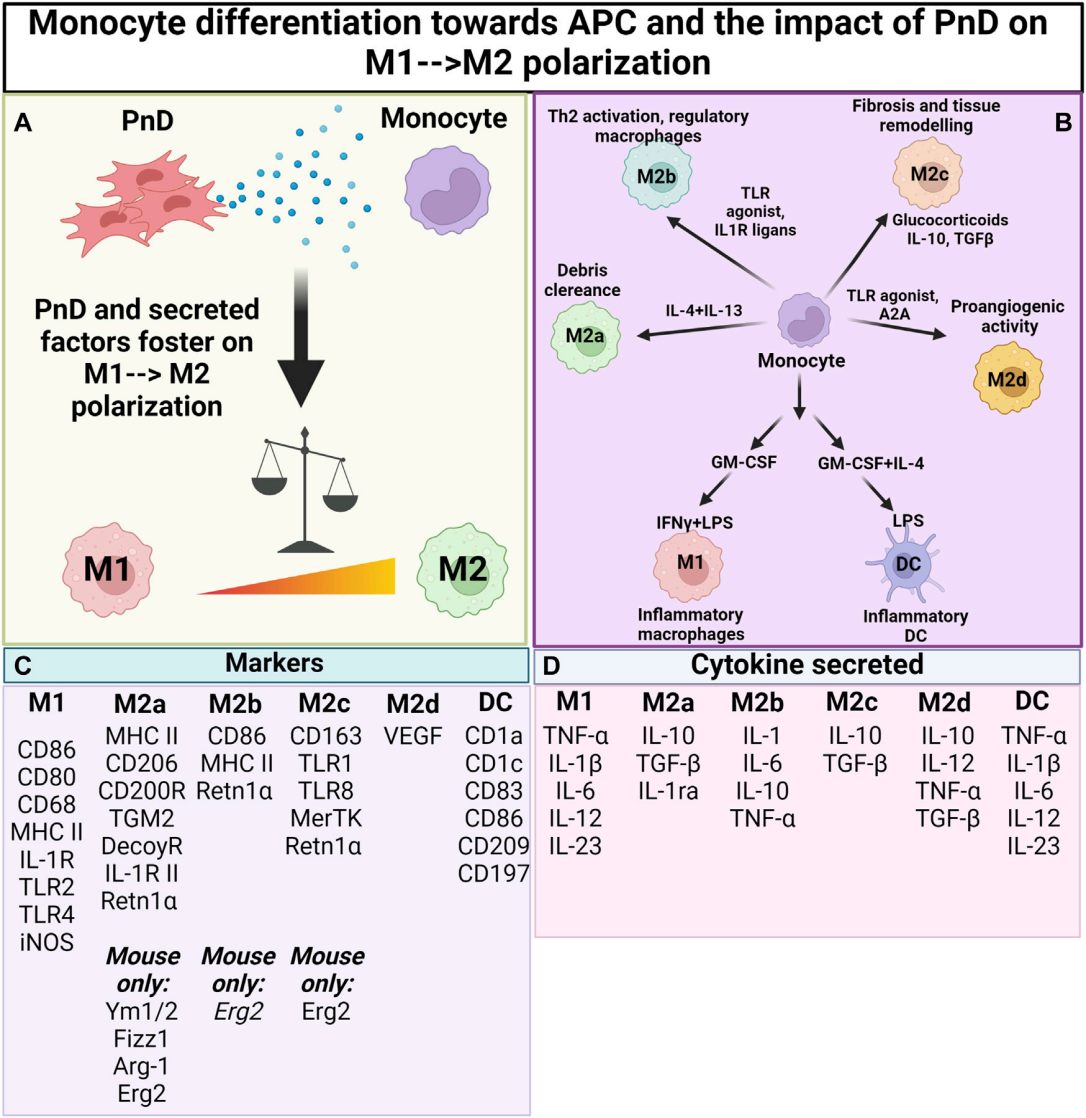
Impact of PnD on monocyte differentiation towards antigen presenting cells. **(A)** Perinatal Derivatives (PnD) and their secreted factors impact monocyte differentiation towards antigen presenting cells fostering the acquisition of phenotype and functional features typical of M2 macrophages. **(B)** Depending on the factors present in the microenvironment monocytes can be discriminated into different subsets of M2 macrophages (M2a, M2b, M2c and M2d) each of them being characterized by peculiar functions. **(C)** Summary table for the markers specific for macrophage and DC subsets. **(D)** Summary table for the cytokines released by the different macrophage/DC subsets. (Created with BioRender.com).

Macrophages and their progenitors, the monocytes, are cells of innate immunity and are involved in the maintenance of tissue homeostasis (Wynn and Vannella, 2016). During tissue injury or infection, macrophages are triggered to phagocytose microbes (Wynn and Vannella, 2016), secrete pro-inflammatory factors that initiate inflammation, and recruit other immune cells to the site of injury/infection. As the insult is cleared, macrophages participate in tissue regeneration by secreting anti-inflammatory/tolerogenic factors that facilitate regenerative processes such as angiogenesis and proliferation, critical for tissue repair and regeneration (Wynn and Vannella, 2016). This functional plasticity of the macrophages has been conceptualized as macrophage polarization, with pro-inflammatory macrophages termed classically activated or M1 macrophages, and anti-inflammatory macrophages termed alternatively activated or M2 macrophages (Mills et al., 2000). Moreover, whilst beyond the scope of this review, M2 macrophages show a high complexity and can be divided into four major types based on their roles: M2a, M2b, M2c and M2d (Murray et al., 2014; Xue et al., 2014) (Figure 2B).

Since macrophages play decisive roles in controlling defence or regenerative immune responses and also are involved in the physiopathology of many diseases, they are the cell type of choice for many research groups in evaluating the immunomodulatory capability of therapeutic cells and their products. In this sense, and similar to the T cells, biological assays employing monocytic cell lines or primary cells should be developed to assess and better characterize the effect of PnD or adult MSC products on these immune cells. In this sense, to set up a biological assay, the choice of a cell line or primary cells is crucial, as it is a compromise between translationality and reproducibility. While primary cells may better reflect the MoA of a product *in vivo*, high variability between donors may hinder the development of a standardized assay, while the opposite is true for cell lines.

In terms of cell lines, human THP-1 monocytic and murine RAW 264.7 macrophage cells, and as primary cells, human peripheral blood monocytes or murine bone marrow-derived monocytes are most frequently used. Primary monocytes can be efficiently differentiated into macrophages (or dendritic cells) using selective cytokine cocktails, typically either containing GM-CSF (+IL-4) or M-CSF. Of note, and highly compromising standardization, most laboratories have their own strategies to prepare, culture and stimulate their monocytic cells or macrophages.

Monocytes and macrophages are most frequently stimulated with interferon (IFN)-γ and LPS to polarize them towards pro-inflammatory M1 macrophages or with IL-4 and IL-13 for obtaining anti-inflammatory M2 macrophages (Figure 2B). In addition, a huge variety of other stimulatory molecules are used for functional activation—including TLR agonists, nucleotide derivatives, glucocorticoids, and antibody-Fc receptor stimulation (Murray et al., 2014). It is however important to note that different polarization programs may be initiated, depending on the stimulus, and macrophages may not polarize solely into M1 or M2 phenotypes (Xue et al., 2014). In order to characterize the macrophage subpopulations, flow cytometric analysis is commonly performed to assess CD80 and CD86 positivity for pro-inflammatory M1 polarisation, and for CD163 or CD206 positivity for anti-inflammatory M2 polarisation. Often, released cytokines are also analysed, with IL-1β and TNF-α considered to be markers of pro-inflammatory M1 macrophage function, and with Arginase (Arg)-1, IL-10, and Retnlα as markers for anti-inflammatory M2 macrophage function (Figures 2C,D).

However, it is noteworthy to mention that many of the murine markers have not been translated to human macrophages and that there are markers that are only present in murine cells while others are only present in human cells (Murray et al., 2014). In this sense, murine macrophages are strong producers of NO in response to LPS stimulation, while human macrophages barely produce NO (Padgett and Pruett, 1992). Moreover, both mouse and human macrophages are able to express Arg-1, but only the latter secrete it (de Boniface et al., 2012). To add to the complexity, even though Arg-1 is usually considered to be an M2 marker, M1 macrophages can also express this enzyme and the same is true for IL-6 in mouse cells (Murray et al., 2014). As a consequence, more than one marker should be assessed to define the macrophage subpopulation. When studying human monocytes and macrophages, typically CD206 is used as a cell surface marker protein reflecting M2 type monocytes and macrophages. In mouse, Erg2 has recently been suggested as a reliable marker for flow cytometric analysis (Jablonski et al., 2015). Likewise, one should realize that the most extreme M1 and M2 macrophage polarisation stages that can be obtained in *in vitro* experiments only rarely occur *in vivo*, where polarisation—and subsequent immune function—is highly dependent on tissue/disease-derived environmental cues and on the interaction with other immune cells (Quarta et al., 2020; 2021a) (Figure 2; Table 3).

**TABLE 3:** Macrophage polarization analysis.

Experimental model	Observed Effects	References
PBMC or purified monocytes	CD80 and CD86 positivity for pro-inflammatory M1 polarisation; CD163 or CD206 positivity for anti-inflammatory M2 polarisation	Murray et al. (2014), Xue et al. (2014)
PBMC or purified monocytes	cytokine analysis where IL-1β and TNF-α are considered to be markers of pro-inflammatory M1 macrophage function, IL-10 is considered a marker of M2 macrophage induction	Murray et al. (2014), Xue et al. (2014)
PBMC or purified monocytes	gene expression analysis for genes canonically expressed by M1 or M2 macrophages like iNOS, Arginase 1, Retn1a	Murray et al. (2014), Xue et al. (2014)
PBMC or purified monocytes	mMurine macrophages are strong producers of NO in response to LPS stimulation, while human macrophages barely produce NO. Both mouse and human macrophages are able to express Arg-1	Padgett and Pruett (1992), de Boniface et al. (2012)
Bone marrow derived macrophages	Erg2 is a new marker for flow cytometry analysis	Jablonski et al. (2015)
RAW 264.7 cells	MSC-EVs have been reported to modulate macrophage phenotypes in several injuries and diseases such as severe asthma	Dong et al. (2021)
Macrophage polarization in a mouse model of bronchopulmonary dysplasia	MSC-EVs have been reported to modulate macrophage phenotypes in several injuries and diseases such as bronchopulmonary dysplasia	Willis et al. (2018)
RAW 264.7 cells	MSC-EVs have been reported to modulate macrophage phenotypes in several injuries and diseases such as skeletal muscle contusion	Luo et al. (2021)
Macrophage polarization in a rat osteochondral defect model	MSC-EVs have been reported to modulate macrophage phenotypes in several injuries and diseases such as cartilage/bone defect	Zhang Chuah et al. (2018)
THP1 cells	MSC-EVs activate TLR4 in a MYD88-dependent pathway through Fibronectin Containing Extra Domain A (FN-EDA)	Zhang et al. (2014b)
RAW 264.7 cells	MSC-EVs inhibit IL-6 secretion in LPS-stimulated macrophages (RAW 264.7 cells)	Pacienza et al. (2019)

Previous studies have noted the immunomodulatory capabilities of MSCs and their secreted EV products on enhancing M2 over M1 macrophage polarization facilitate tissue repair (Zhang B. et al., 2018; Willis et al., 2018; Luo et al., 2021; Chuah et al., 2022). In this sense, MSC-EVs have been reported to modulate the macrophage phenotypes in several injuries and diseases such as severe asthma (Dong et al., 2021), bronchopulmonary dysplasia (Willis et al., 2018), skeletal muscle contusion (Luo et al., 2021) and cartilage/bone defects (Zhang B. et al., 2018). This implicates the role of macrophages as the therapeutic target of MSCs and their EVs in tissue repair. Consistently, depletion of macrophages abolished the therapeutic effects of MSCs in tissue repair (Luo et al., 2021). Thus, these findings support and should encourage the use of macrophages for the development of *in vitro* cell-based assays to assess the immunomodulatory capabilities also of PnD products, especially in pathologies where this immune cell type plays major roles. Nevertheless, referring to the tissue-dependent context where macrophages reside, most of the currently applied macrophage polarisation studies (and influence of potential modulators thereon) lack a tissue-specific context. For example, it has recently been demonstrated using murine iPSC-derived macrophages—and further confirmed *in vivo*—that both M1 and M2 polarisation is highly influenced by neural environments (Quarta et al., 2019; Quarta et al., 2021b).

Regarding the modulation of monocyte/macrophage polarisation by PnD and adult MSC products, like that of T cell polarisation, the observed effect is dependent on the stimuli applied to the cells. For instance, it was previously reported that MSC-EVs activate TLR4 in a MYD88-dependent pathway in THP-1 cells through Fibronectin Containing Extra Domain A (FN-EDA) being present in the secretome (Zhang et al., 2014). Unlike LPS which activates TLR4 in the same pathway, MSC-EVs did not induce the expression of pro-inflammatory cytokines, but instead induce the expression of anti-inflammatory cytokines, such as IL-10. This phenomenon was reproducible in primary human and mouse monocytes. Therefore, the design and implementation of an *in vitro* macrophage polarization assay with the relevant functional endpoints should allow the exploration of PnD and adult MSC products on macrophage polarization. Considering their impact on macrophages, as one of the key immunomodulatory functions of PnD and adult MSC products, such assays should also enable the search for critical PnD and adult MSC attributes that manifest their therapeutic potency (Figure 2A).

Of note, an *in vitro* assay has recently been established using RAW 264.7 cells for the assessment of anti-inflammatory activities of given MSC-EV products. The assay documents the ability of MSC-EV preparations to inhibit IL-6 secretion in LPS-stimulated macrophages. Interestingly, this RAW 264.7 cell-based assay showed that different MSC-EV batches vary in their macrophage polarisation abilities, and that its activity predictions correlate with their documented *in vivo* functions obtained in a mouse model of LPS-induced systemic inflammation (Pacienza et al., 2019). Furthermore, as with the activation, suppression or polarisation of T cells, developing novel assays in which the downstream effect of polarised macrophages (e.g., by addition of PnD or adult MSC products) is investigated on T cell function is highly advisable. Such studies could help to elucidate cross talks between the innate and adaptive immune systems whose dysregulation can result in the adoption of inflammatory and autoimmune diseases (Figure 2A; Table 3).

## How to identify the right assay

Due to the high number of variables and the fact that each disease may require specific therapeutic activities, it is hard to provide any concrete recommendations for a certain assay type. Considering product heterogeneity, it may be best for the establishment and validation of appropriate assays, if products manufactured in the same standardized manner are available that differ in their clinical or preclinical potency to improve symptoms in a given disease model. Assay candidates should provide the same prediction for an assumed MoA as observed *in vivo*. To this end, as elaborated in a recent position paper on potency testing of MSC-EV products (Gimona et al., 2021), it might be that the MoA requires the combined action of different biological activities, the so called MoA attributes. If different MoA attributes are required for defining the therapeutic potency of PnD and adult MSC products, an array matrix consisting of several potency assays may become required for appropriate potency testing of respective drugs (https://www.fda.gov/media/79856/down) (Chinnadurai et al., 2018; Gimona et al., 2021).

When using primary cells as test cell type, donor-to-donor variations in the reactivity of the assay cells, e.g., the T cells and macrophages, need to be considered. Cell lines might be altered so substantially that they do no longer allow monitoring of the given cell activity. Moreover, knowing the pathophysiology of a given disease and the role that each immune cell plays is critical to choose the right cell type to assess the activity of a given PnD and adult MSC product. Having identified an *in vitro* assay reflecting the *in vivo* potency of the PnD or adult MSC products, it needs to be considered whether or not the assay can be qualified as a potency assay. Of note, functional assays providing information about the potency of given PnD or adult MSC products are not automatically potency assays. The term Potency Assay is a regulatory authority term and deciphers an assay which had been standardized and qualified to be very reproducible. As elaborated in a recent white paper (Gimona et al., 2021), a potency assay needs to be designed and to predict the therapeutic effectiveness of the drug substance in accordance with the International Council for Harmonisation of Technical Requirements for Pharmaceuticals for Human Use (ICH) guidelines. Due to the variation of the biology of primary cells, setting up potency assays based on primary cells is very challenging (Gimona et al., 2021). Here, cell lines might be the better choice, providing that they are able to recapitulate the MoA of the PnD or adult MSC products that are required to alleviate the disease. Although still in its infancy, replacement of monocytic cell lines with standardized batches of human iPSC-derived monocytes/macrophages may well become part in future potency assay development. Setting up the appropriate potency assays remains a major challenge in the field of therapeutic development of PnD and adult MSC products. Although the use of animal cells for testing of human therapeutics remains controversial, murine immune cell lines such as the RAW 264.7 cells remain to date widely be used for testing the immunomodulatory potential of MSCs and their products (Zampetaki et al., 2004; Pacienza et al., 2019; Malvicini et al., 2022).

## Conclusion

As exemplified by the decision of the US FDA to not provide market approval to remestemcel-L, potency testing of cellular and secretome based drugs, including PnD and adult MSC products, is a central task for the future. Although many functional activities can be read out in available assays, it is necessary to confirm that these activities reflect actual MoA attributes that are required to reduce pathophysiological symptoms in given diseases. A functional assay reflecting such MoA attributes, and thus the potency of PnD and adult MSC products, is not automatically a potency assay. A potency assay has to fulfill several regulatory requirements. We envisage that the establishment of appropriate potency tests will remain a major challenge in the cell and EV-based therapeutic field.
